# Soft tissue stabilization of the hinge position in medial closed wedge distal femoral osteotomy: an anatomical study

**DOI:** 10.1186/s12891-022-06078-y

**Published:** 2022-12-19

**Authors:** Taiga Oda, Akira Maeyama, Ichiro Yoshimura, Tetsuro Ishimatsu, Kotaro Miyazaki, Katsuro Tachibana, Kengo Yoshimitsu, Takuaki Yamamoto

**Affiliations:** 1 Department of Orthopedic Surgery, Faculty of Medicine, Fukuoka University, 7-45-1 Nanakuma, Jonan-Ku, Fukuoka, 814-0180 Japan; 2 Department of Anatomy Faculty of Medicine, Fukuoka University, 7-45-1 Nanakuma, Jonan-Ku, Fukuoka, 814-0180 Japan; 3 Department of Radiology Faculty of Medicine, Fukuoka University, 7-45-1 Nanakuma, Jonan-Ku, Fukuoka, 814-0180 Japan

**Keywords:** Medial closed wedge distal femoral osteotomy, Lateral hinge, Periosteum, Capsule, Attachment

## Abstract

**Background:**

Soft tissue has an important role in stabilizing the hinge point of medial closed wedge distal femoral osteotomy (MCWDFO). However, there are conflicting data on the soft tissue anatomy around the hinge point of MCWDFO and, therefore, further anatomical data are needed. The purposes of the study were to: 1) anatomically analyze the soft tissue around the hinge point of MCWDFO; 2) radiologically define the appropriate hinge point to prevent an unstable hinge fracture based on the result of the anatomical analysis; and 3) histologically analyze the soft tissue based on the result of the anatomical analysis.

**Methods:**

In 20 cadaveric knees, the capsule attachment of the distal lateral side of the femur was marked with a radiopaque ball bearing. A digital planning tool was used to calculate the area of the marked capsule attachment around the ideal hinge point of MCWDFO on radiographs. The soft tissue around the hinge point was histologically examined and the periosteal thickness was measured and visualized graphically. The graph and radiograph were overlayed using image editing software, and the appropriate hinge position was determined based on the periosteal thickness.

**Results:**

As a result, the periosteal thickness of the distal lateral femur tended to rapidly decrease from the metaphyseal region toward the diaphyseal region. The overlayed graph and radiograph revealed that the periosteal thickness changed in the region corresponding to the apex of the turning point of the femoral metaphysis in all cases.

**Conclusions:**

In conclusion, the periosteum might support the hinge of MCWDFO within the area surrounded by the apex of the turning point of the femoral metaphysis and the upper border of the posterior part of the lateral femoral condyle.

## Background

Valgus deformity of the knee is rarer than varus deformity and is present in 15% of all knees requiring total knee arthroplasty [1, 2]. Most cases of valgus deformity are caused by the femur; therefore, the validity of distal femoral osteotomy for valgus knee has recently been reported [3, 4]. One distal femoral osteotomy technique is medial closed wedge distal femoral osteotomy (MCWDFO), which is widely used to correct valgus deformity in young active patients [5, 6].

The reported survival rate of MCWDFO ranges from 64.0% to 89.8% at 10 years postoperatively, and an acceptable clinical outcome is expected once the osteotomy site is united [7, 8]. However, unstable lateral hinge fracture is considered an important cause of instability at the osteotomy site and may lead to displacement at the osteotomy site, broken screws, malunion or nonunion, and loss of correction [9–12]. Therefore, it is important to identify the optimal hinge point that prevents unstable lateral hinge fracture.

In medial opening wedge high tibial osteotomy, the region from the tip of the fibular head and the circumference line of the fibular head is defined as the “safe zone” in which the lateral capsule of the femorotibial joint capsule–periosteal sleeve and the tibialis anterior muscle work as soft tissue stabilizers, reducing the likelihood of lateral cortical disruption [13, 14]. Moreover, in medial opening wedge supramalleolar osteotomy, the plane of the proximal one-third of the intrasyndesmosis is defined as the “safe zone” in which a thick periosteum and a large, tight interosseous tibiofibular ligament complex stabilize the osteotomy site [15].

Several studies have been performed in an attempt to define the “safe zone” for MCWDFO in which the risk of unstable hinge fracture is minimized and there is no risk of cutting into the joint space in the distal direction. An anatomical study of the lateral distal femur showed the presence of the gastrocnemius lateral head (GLH), periosteum, and capsule around the hinge point of MCWDFO. The study suggested that these soft tissues play a small role in the stability of the osteotomy hinge [12]. However, no specific reason or details were given for this conclusion. One study showed that the GLH attaches to the upper border of the lateral femoral condyle and stabilizes the hinge point of MCWDFO [9]. However, the other possible soft tissue stabilizers of lateral hinge fracture might have been stripped away during anatomical dissection, and thus the impact of these soft tissues was not fully considered. Furthermore, another study revealed a different origin of the GLH near or at the supracondylar process [16], suggesting that the GLH alone may not provide sufficient support for the hinge.

These conflicting reports show that there is a need for further anatomical data regarding the soft tissue around the hinge point of MCWDFO. We hypothesized that the “safe zone” in MCWDFO can be defined by investigating the soft tissue around the hinge point. The purposes of the study were to: 1) anatomically analyze the soft tissue around the hinge point of MCWDFO; 2) radiologically define the appropriate hinge point to prevent an unstable hinge fracture based on the result of the anatomical analysis; and 3) histologically analyze the soft tissue based on the result of the anatomical analysis.

## Methods

The present study involved 30 knees from 30 cadavers donated to our institute from July 2016 to July 2019. Six knees were excluded because of severe osteoarthritis, flexion contracture, and/or previous knee surgery, and four knees were used for a pilot study. As a result, 20 knees from 20 cadavers were evaluated in this study. The characteristics of the cadavers are listed in Table 1. The cadavers were fixed with 37% formalin and preserved in 95% ethanol. Before death, all individuals had provided informed consent for the donation of their body for anatomical education and study. Our study received ethical approval from our institutional review board prior to study commencement.

**Table 1:** Cadaver characteristics and measured femoral widths and lengths

	**Mean ± SD**	**Range**
Right: Left	14: 6	
Male: Female	10: 10	
Age (y)	78.8 ± 9.3	61–91
Height (cm)	159.3 ± 8.6	140–174
ML dimension of distal femur (cm)	83.7 ± 6.0	74.0–95.2
AP dimension of distal femur (cm)	62.6 ± 3.9	54.7–68.7
Length of femur (cm)	40.2 ± 3.7	33.0–46.2

*SD* Standard deviation, *ML* Mediolateral, *AP* Anteroposterior

### Anatomical analysis of soft tissue around hinge point of MCWDFO

**Fig. 1:**
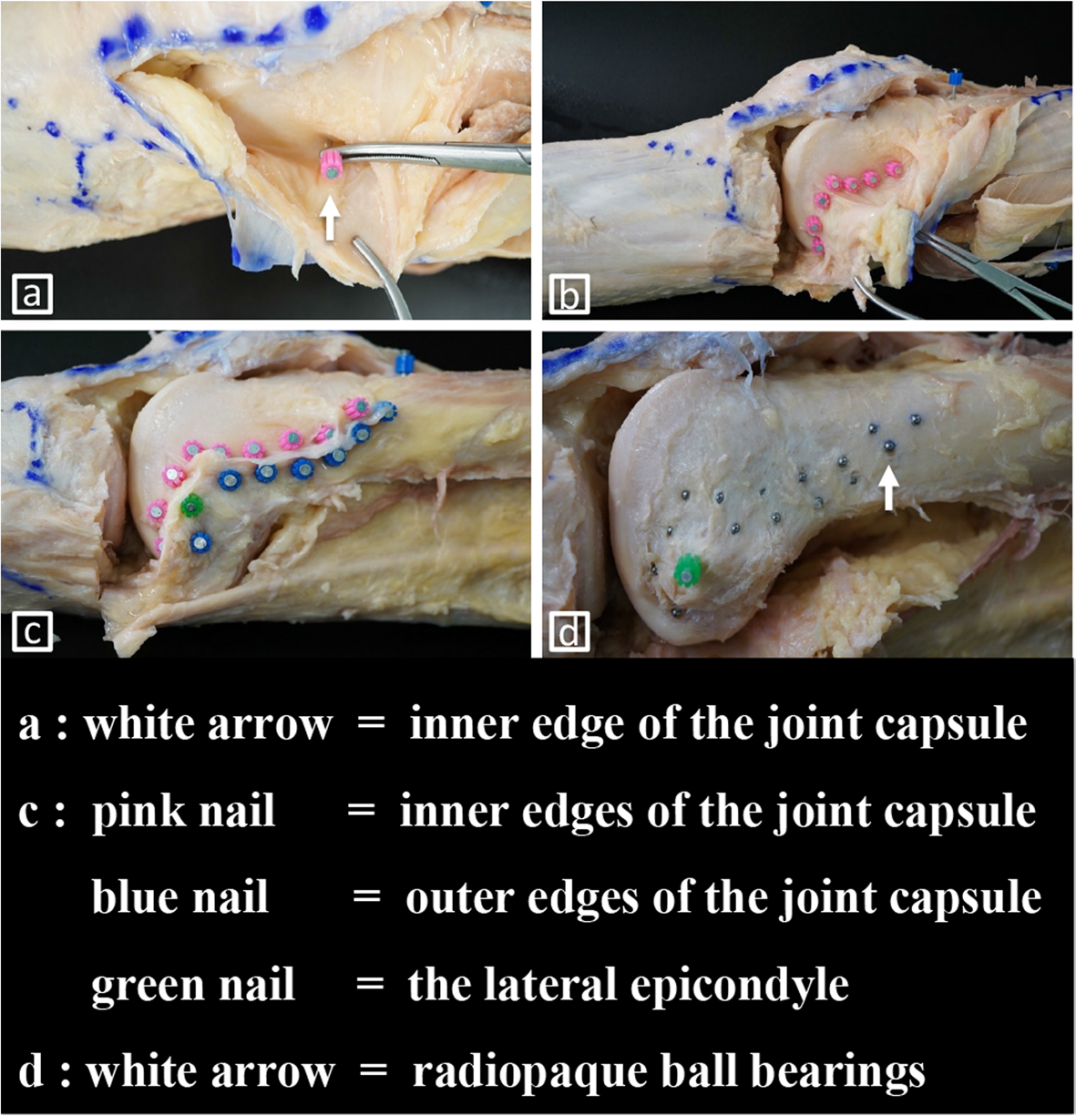
Dissection of the lateral aspect of the distal femur and insertion of ball bearings. **a** The attachment of the joint capsule to the distal femur was confirmed by tensioning with minimal force. **b**, **c** The margins of the inner and outer edges of the joint capsule and the lateral epicondyle were marked with colored nails. **d** Holes (2 mm-diameter) were drilled into the marked cortical bone and 2 mm-diameter radiopaque ball bearings were inserted

Two knees were used to investigate the GLH attachment as a pilot study. A 30- × 30-cm skin incision was made on the distal lateral side of the knee joint and the skin was excised with a scalpel. The lateral parapatellar approach was used for capsulotomy. Another skin incision was then made in the center of the quadriceps femoris and the ligamentous layers were detached from the proximal to distal direction to expose the entire joint capsule. The iliotibial band from Gerdy’s tubercle and the biceps femoris tendon from the fibular head were then dissected and inverted, respectively. The attachment of the joint capsule to the distal femur was confirmed by tensioning with minimal force and the margins of the inner and outer edges of the joint capsule were marked with colored nails (Fig. 1a-c). Drill holes (2 mm in diameter) were made into the marked cortical bone and 2-mm-diameter radiopaque ball bearings (G25 AISI 52,100 chrome steel; Uxcell, Hong Kong, China) were inserted (Fig. 1d).

**Fig. 2:**
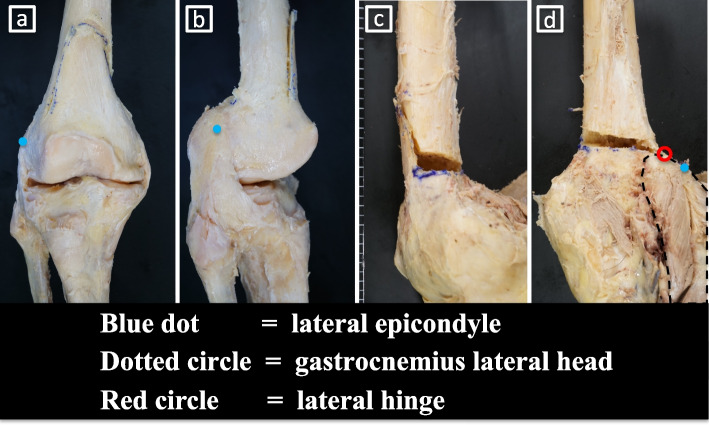
Biplanar MCWDFO. Biplanar MCWDFO was performed with two knees according to the osteotomy level recommended by Kim et al. [9]. **a** Anteroposterior view. **b** Lateral view. **c** Medial view. **d** Posteroanterior view. The lateral head of the gastrocnemius muscle was attached distal to the osteotomy line on the posterior side of the femur

The mediolateral (ML) and anteroposterior (AP) dimensions of the femoral condyle and the total length of the femur were then measured at 0.1-mm intervals using a digital caliper (IP54 digital caliper; GAWOOW, China). Lastly, as a pilot study, biplanar MCWDFO was performed with two knees according to the osteotomy level recommended by Kim et al. (Fig. 2a-d) [9].

### Radiological analysis of capsule attachment and periosteal thickness

**Fig. 3:**
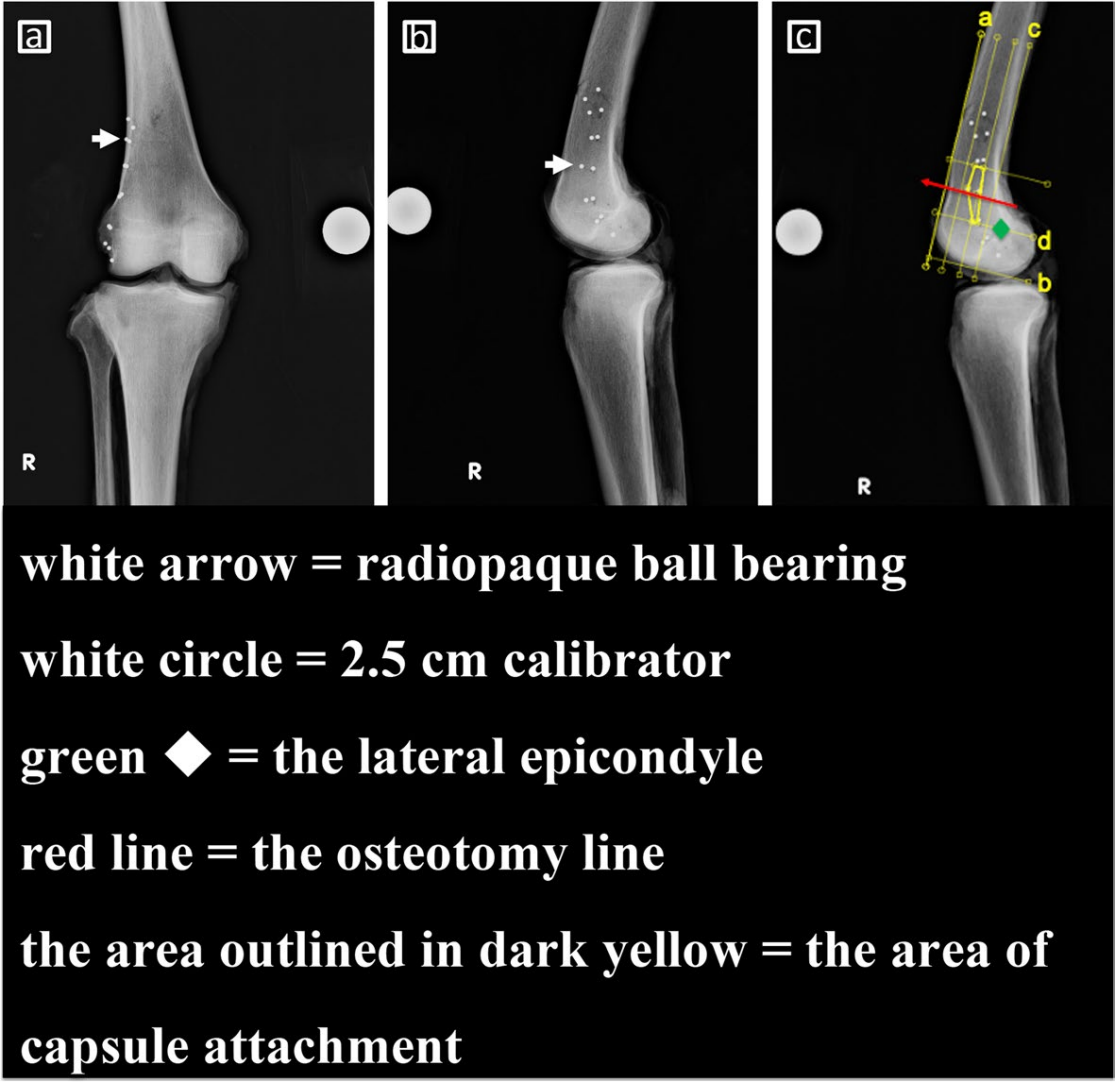
Radiography of the marked capsule attachment of the lateral distal femur. **a** frontal view, (**b**) sagittal view, (**c**) area of capsule attachment around the hinge point of medial closed wedge distal femoral osteotomy (MCWDFO). Line a is parallel to the anterior surface of the femoral shaft. Line b is perpendicular to line a and passes through the most distal part of the lateral distal femoral condyle. Line c is parallel to line a and in contact with the posterior part of the distal femoral shaft. Line d is parallel to line b and passes through the lateral epicondyle. A rectangle centered on the osteotomy line is created by lines a, c, and d. The rectangular area is divided into three regions: front; middle; and rear. The area of capsule attachment in the middle and rear regions is calculated using a digital planning tool

**Fig. 4:**
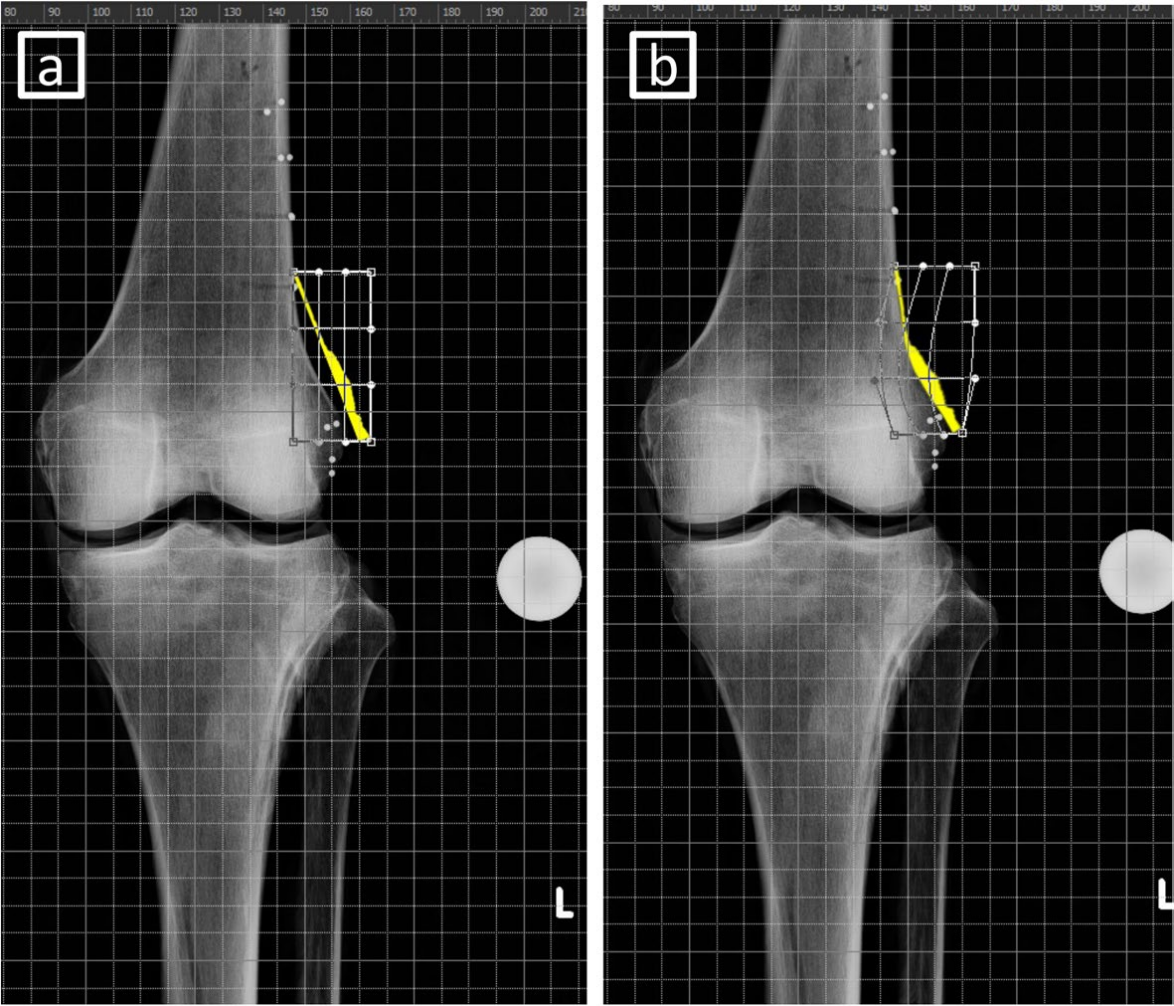
Methods of overlaying the graph of periosteum with X-ray image by image editing software. **a** Before the deformation. The most distal part of the graph was placed at the level of the lateral epicondyle and parallel to the curved part of the femoral condyle. **b** After the deformation. The graph was deformed along the curved part of the metaphysis of the distal femur

Frontal and sagittal radiographs of all amputated knees were taken with a 2.5-cm calibrator. The radiographic conditions were 70 kV, 7 mAs, and distance of 120 cm. The marked area of the joint capsule attachment around the hinge point of MCWDFO was calculated using a digital planning tool (Ortho Planner Pro; Toyo Technica, Tokyo, Japan) (Fig. 3a-c). The sagittal radiograph and the graph of the periosteal thickness were overlayed using image editing software (Adobe Photoshop CS6; Adobe Inc., San Jose, CA, USA). First, the graph was calibrated with a 2.5-cm calibrator. Next, the most distal part of the graph was placed at the level of the lateral epicondyle and parallel to the curved part of the femoral condyle. The warp tool of Adobe Photoshop was then used for the deformation. To minimize scale fluctuation due to deformation, deformation was performed only in the same axial direction using a grid, and the distance of movement between the grids was adjusted to be equal. Finally, the graph was recalibrated to adjust the distance (Fig. 4a-b).

### Histological examination of soft tissue

**Fig. 5:**
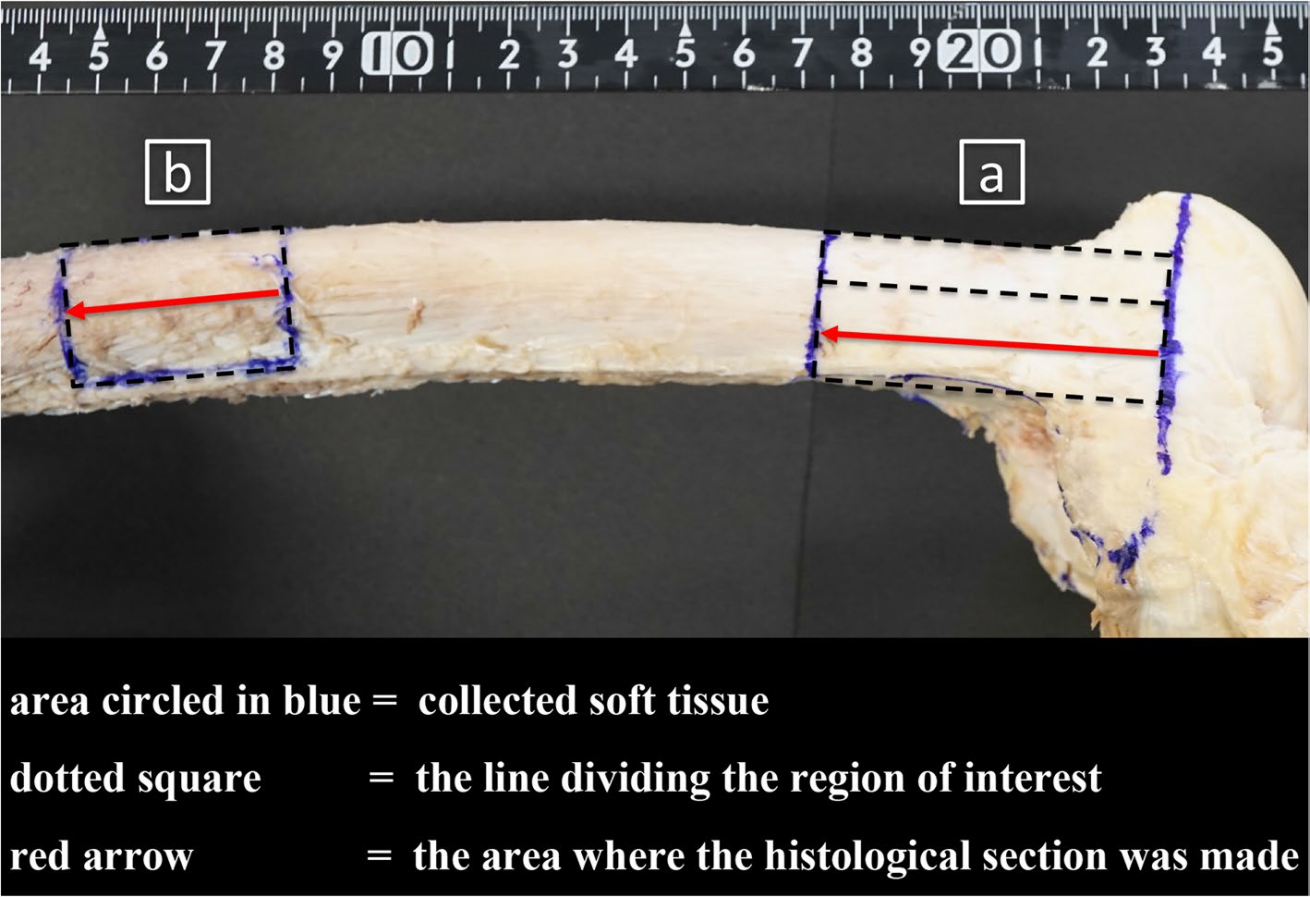
Histological section of lateral femur. **a** Soft tissue was collected (6 cm-wide section) including bone from the lateral epicondyle to the proximal direction. The histological section was made passing through 2/3 of the divided area where the hinge of MCWDFO is usually made. **b** Soft tissue of the femoral shaft including bone was collected (3 cm-wide section) for comparison. The histological section was made passing through the middle of the divided area

Soft tissue was collected in 6-cm-wide sections, including bone from the lateral epicondyle to the proximal direction. The soft tissue was divided into three regions (anterior, middle, and posterior), and the histological sections were made passing between the middle and posterior regions where the hinge of MCWDFO is usually made. Soft tissue and bone of the femoral shaft were collected in 3-cm-wide sections for comparison. The histological sections were made passing through the middle of the divided area (Fig. 5). Blocks comprising the metaphysis of the femur and periosteum were obtained. The blocks were fixed in 8% formalin and decalcified in a solution containing aluminum chloride, hydrochloric acid, and formic acid, as reported previously [17]. The blocks were then embedded in paraffin and sliced into 5-μm sections. The sections were stained with hematoxylin and eosin stain (HE) and Masson’s trichrome stain (MT), as described in a previous report [18]. The thickness of the stained periosteum was measured using an electron microscope (Olympus BX50; Olympus, Tokyo, Japan) at intervals of 500 μm from the distal to proximal direction. The electronic microscopic images were recorded with a digital camera for microscopes (Olympus DP27-B; Olympus). The results of the measured periosteal thickness were visualized graphically.

### Statistical analysis

Statistical analysis was performed using SPSS version 23.0 software (IBM Corp. Armonk, NY, USA). All continuous variables are presented as mean ± standard deviation. Correlations between continuous variables were analyzed using Pearson’s correlation model. Statistical significance was defined as *p* < 0.05.

## Results

### Anatomy of lateral side of the distal femur

The ML dimension, AP dimension, and length of the femurs are shown in Table 1.

**Fig. 6:**
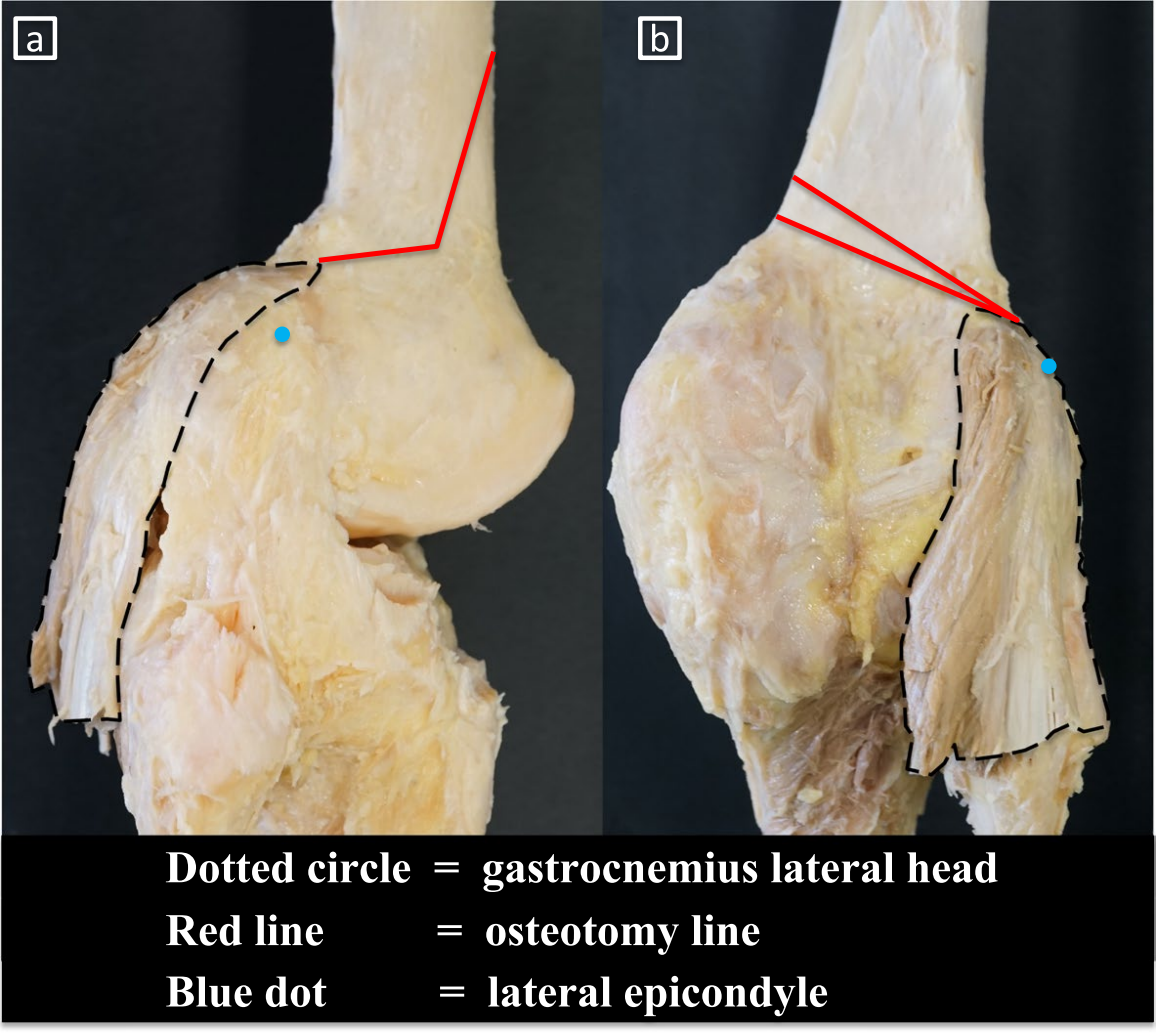
Dissection of the gastrocnemius lateral head. **a** lateral view. **b** posterior view. The GLH was attached to the posterolateral side of the femur; however, the GLH was attached distally and posteriorly to the hinge and seemed to play a small role in stabilizing the hinge point

**Fig. 7:**
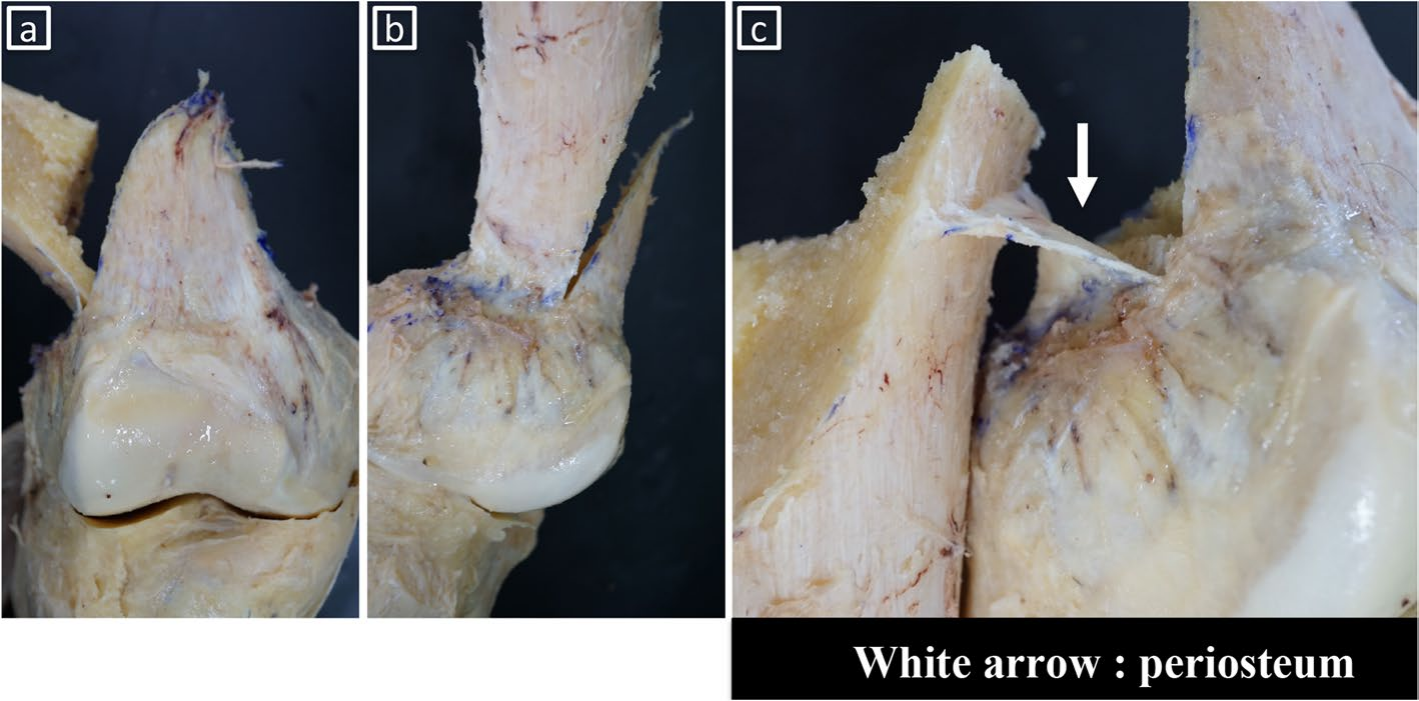
Periosteum around the hinge point of medial closed wedge distal femoral osteotomy. **a** antero-posterior view. **b** lateral view. **c** the periosteum remains without tearing even after the hinge is manually fractured

Our pilot study showed the presence of the GLH on the posterolateral side of the distal femur (Fig. 6a-b). Dissection showed the joint capsule and periosteum directly attached at the lateral side of the distal femur. The joint capsule was fragile enough to be easily detached during dissection. Removal of the joint capsules revealed that the periosteum of the lateral side of the distal femur was thicker than that of the diaphysis. Biplanar MCWDFO was subsequently performed with two knees and even after the hinge was fractured and displaced with maximum manual force, the periosteum remained intact without tearing (Fig. 7a-c).

### Radiological analysis of the area of capsule attachment

**Fig. 8:**
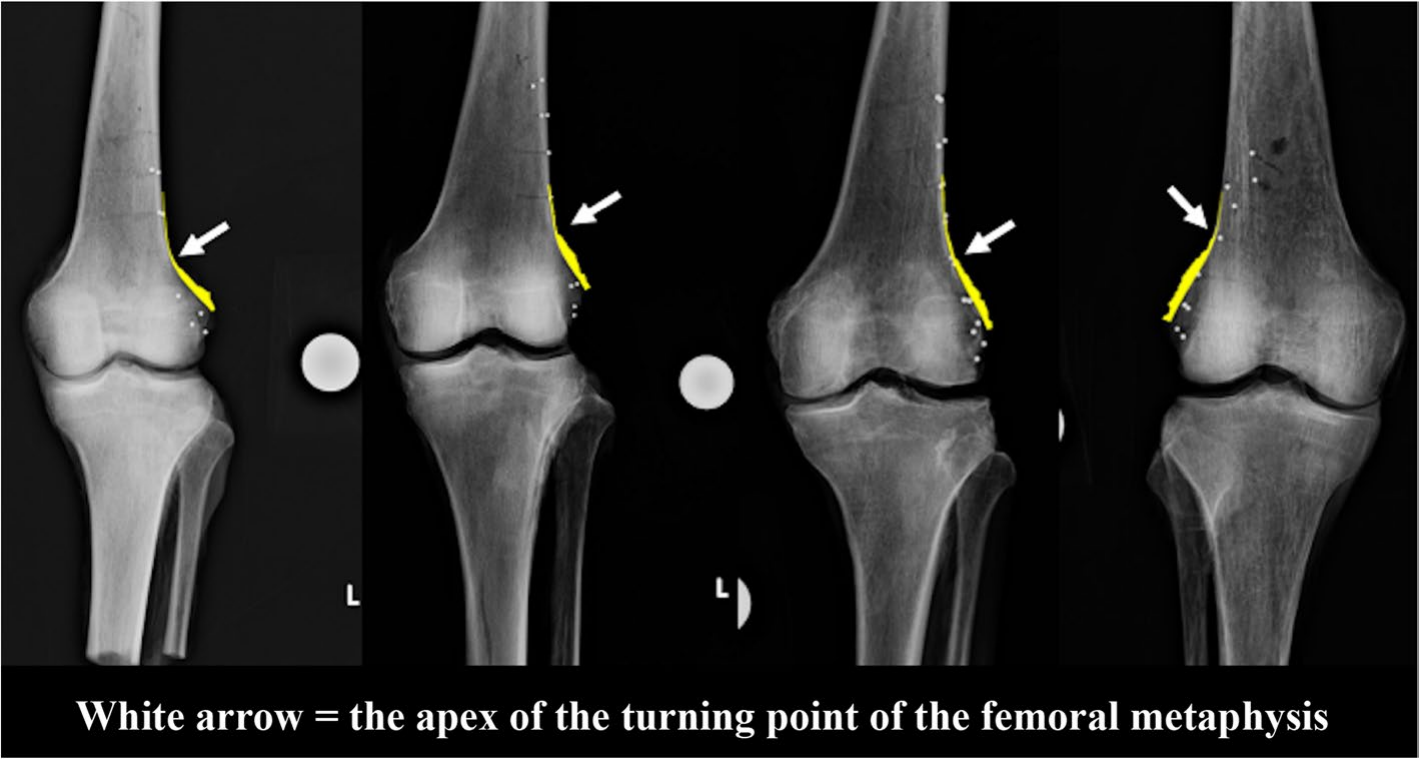
Representative examples of overlayed images created by image editing software. The periosteal thickness changes at the part corresponding to the apex of the turning point of the femoral metaphysis in all cases

The area of marked capsule attachment around the ideal hinge point of MCWDFO was 206.8 ± 31.7 mm^2^ (range, 164.3–278.1 mm^2^). Four representative examples of overlayed images of periosteum are shown in Fig. 8. The periosteal thickness changed at the region corresponding to the apex of the turning point of the femoral metaphysis in all cases.

### Histological examination of soft tissue

**Fig. 9:**
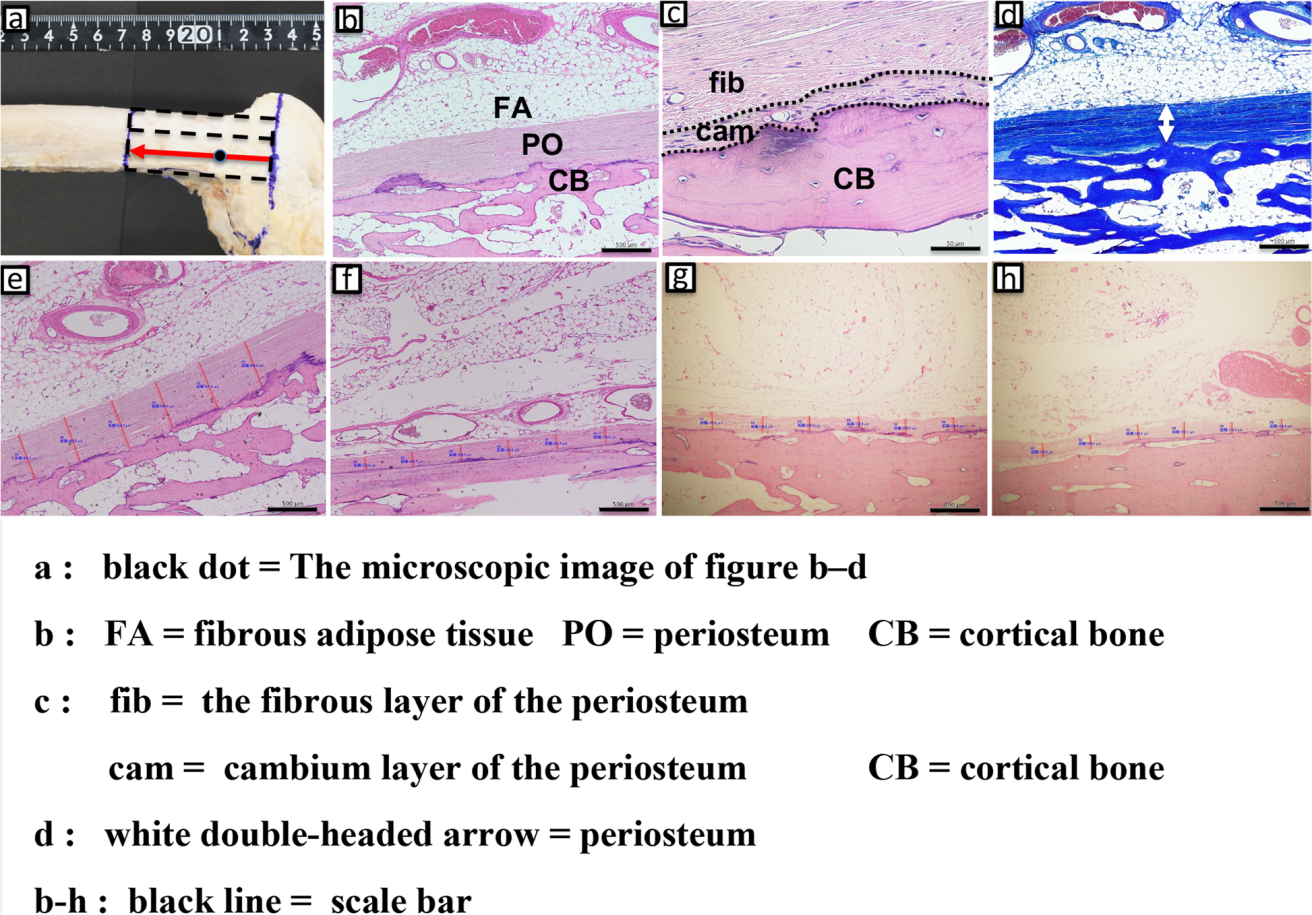
The histological findings of the lateral femur. **a** Creation of the histological section as described in Fig. 5. The microscopic findings of the marked area are shown in b–d. **b** Hematoxylin and eosin (HE) staining showing CB, PO, and FA (HE, × 4. scale bar: 500 μm). The low-powered HE-stained image shows the presence of a continuous layered structure in contact with the bone cortex. Sparse FA was present between the synovial surface of the joint cavity and the PO. **c** HE staining showing the fib, cam, and CB (HE, × 40. scale bar: 50 μm). **d** The layered structure is equally and deeply stained blue by Masson's trichrome (MT) stain and maintains its structure while gradually thinning from the distal to proximal direction (MT, × 4. scale bar: 500 μm). Therefore, these layered structures were considered to be the PO. **e**, **f** Periosteal thickness of the metaphyseal region on the (**e**) distal side and (**f**) proximal side (HE, × 4. scale bar: 500 μm). The periosteum gradually becomes thinner from the distal to the proximal side. **g**, **h** Periosteal thickness of the diaphyseal region on the (**g**) distal side and (**h**) proximal side (HE, × 4. scale bar: 500 μm). The periosteum of the diaphysis is relatively uniform and thin compared with that of the metaphysis

**Fig. 10:**
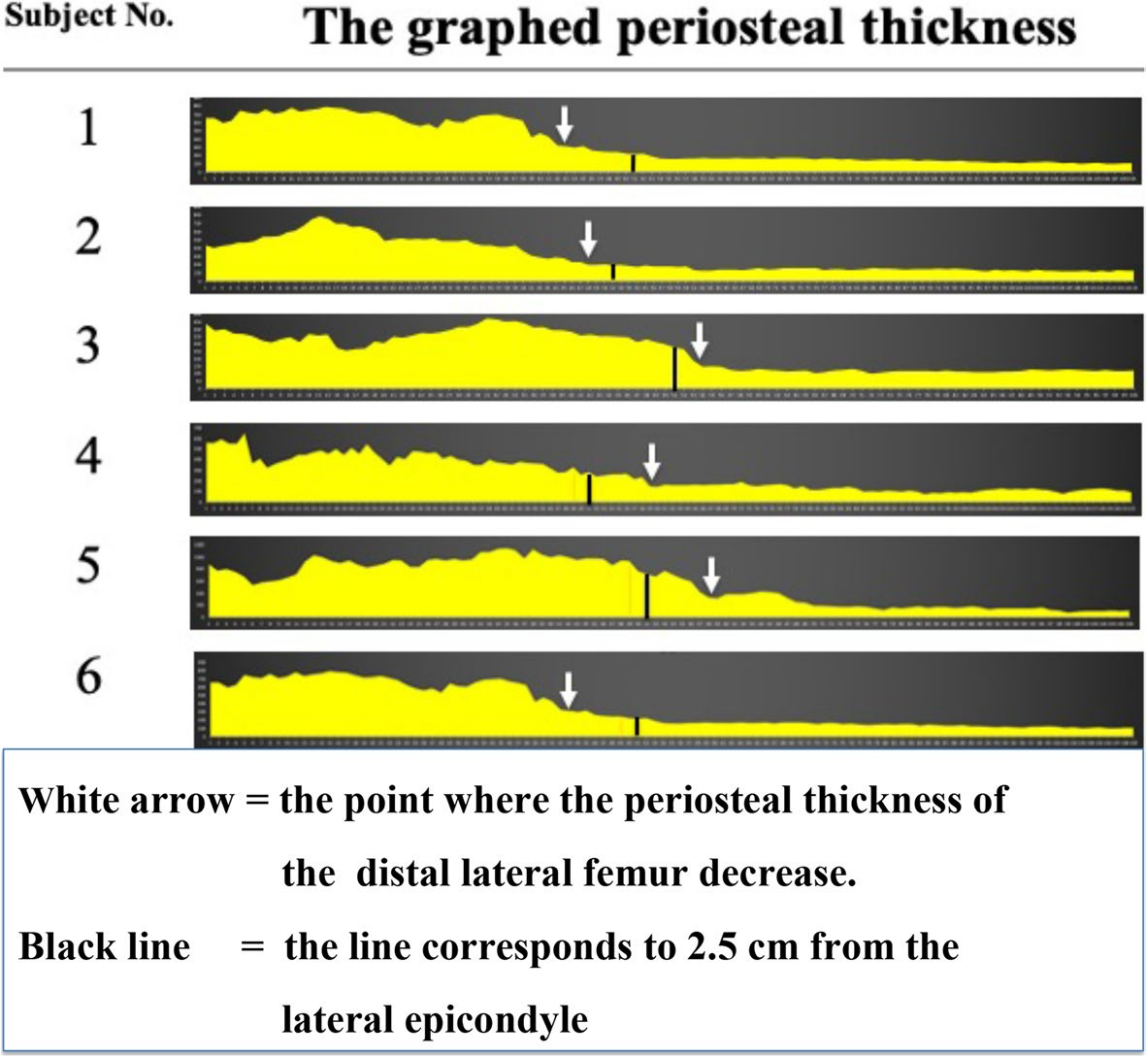
Representative examples of the graphed periosteal thickness. Left side = distal side, right side = proximal side. The periosteal thickness of the distal lateral femur tended to rapidly decrease from the metaphyseal region toward the diaphyseal region

The histological findings of the lateral femur are shown Fig. 9a-h. The measured periosteal thicknesses of the metaphysis and diaphysis at 500-µm intervals were 308.4 ± 93.7 μm (range, 191.2–556.6 μm) and 91.1 ± 12.5 μm (range, 73.7–123.5 μm), respectively. The graphical results of periosteal thickness revealed that the thickness tended to rapidly decrease at a certain distance from the proximal direction, starting from the lateral epicondyle. Six representative examples are shown in Fig. 10.

### Statistical analysis

The area of capsule attachment was significantly related to age (*p* = 0.008, *r* = 0.553), the AP dimension of the femur (*p* < 0.001, *r* = 0.817), the total length of the femur (*p* < 0.001, *r* = 0.837), and height (*p* < 0.001, *r* = 0.793) (Table 2).

**Table 2:** Area of capsule attachment and its relationship to distal femoral dimension

Area of capsule attachment around the hinge point of MCWDFO							
	**Mean ± SD**			**Range**			
**Area of capsule attachment (mm**^**2**^**)**	206.8 ± 31.7			164.3–278.1			
**Relationship between area of capsule attachment and distal femoral dimension**							
		**Age**	**Sex**	**Femur ML**	**Femur AP**	**Femur length**	**Height**
**Area of capsule attachment (mm**^**2**^**)**	***P*** **value**	.008	.036	.266	< .01	< .01	< .01
	***r*** **value**	.553	-.461	.248	.817	.837	.793

*MCWDFO* Medial closed wedge distal femoral osteotomy, *SD* Standard deviation, *ML* Mediolateral, *AP* Anteroposterior

Correlations of the cadaver characteristics with the periosteal thickness of the metaphysis and diaphysis are shown in Table 3. In the region of the metaphysis, the periosteal thickness was significantly related to the AP dimension of the femur (*p* = 0.010, *r* = 0.538), the total length of the femur (*p* = 0.009, *r* = 0.543), and height (*p* = 0.030, *r* = 0.462), but not to age (*p* = 0.050, *r* = 0.422) or the ML dimension of the femur (*p* = 0.782, *r* =  − 0.063). In the region of the diaphysis, the periosteal thickness was significantly related to age (*p* = 0.002, *r* = 0.627), the AP dimension of the femur (*p* < 0.001, *r* = 0.710), the total length of the femur (*p* < 0.001, *r* = 0.751), and height (*p* < 0.001, *r* = 0.786), but not to the ML dimension of the femur (*p* = 0.900, *r* =  − 0.028).

**Table 3:** Correlations of cadaver characteristics and distal femoral dimensions with periosteal thickness of metaphysis and diaphysis

**Metaphysis**		**Age**	**Sex**	**Femur ML**	**Femur AP**	**Femur length**	**Height**
**Average thickness of periosteum**	***P* value**	.050	.032	-.063	< .01	< .01	.03
	***r* value**	.422	-.468	.782	.538	.543	.462
**Diaphysis**		**Age**	**Sex**	**Femur ML**	**Femur AP**	**Femur length**	**Height**
**Average thickness of periosteum**	***P* value**	.020	.357	.900	< .01	< .01	< .01
	***r* value**	.627	-.212	-.028	.710	.751	.786

*ML* Mediolateral, *AP* Anteroposterior

## Discussion

**Fig. 11:**
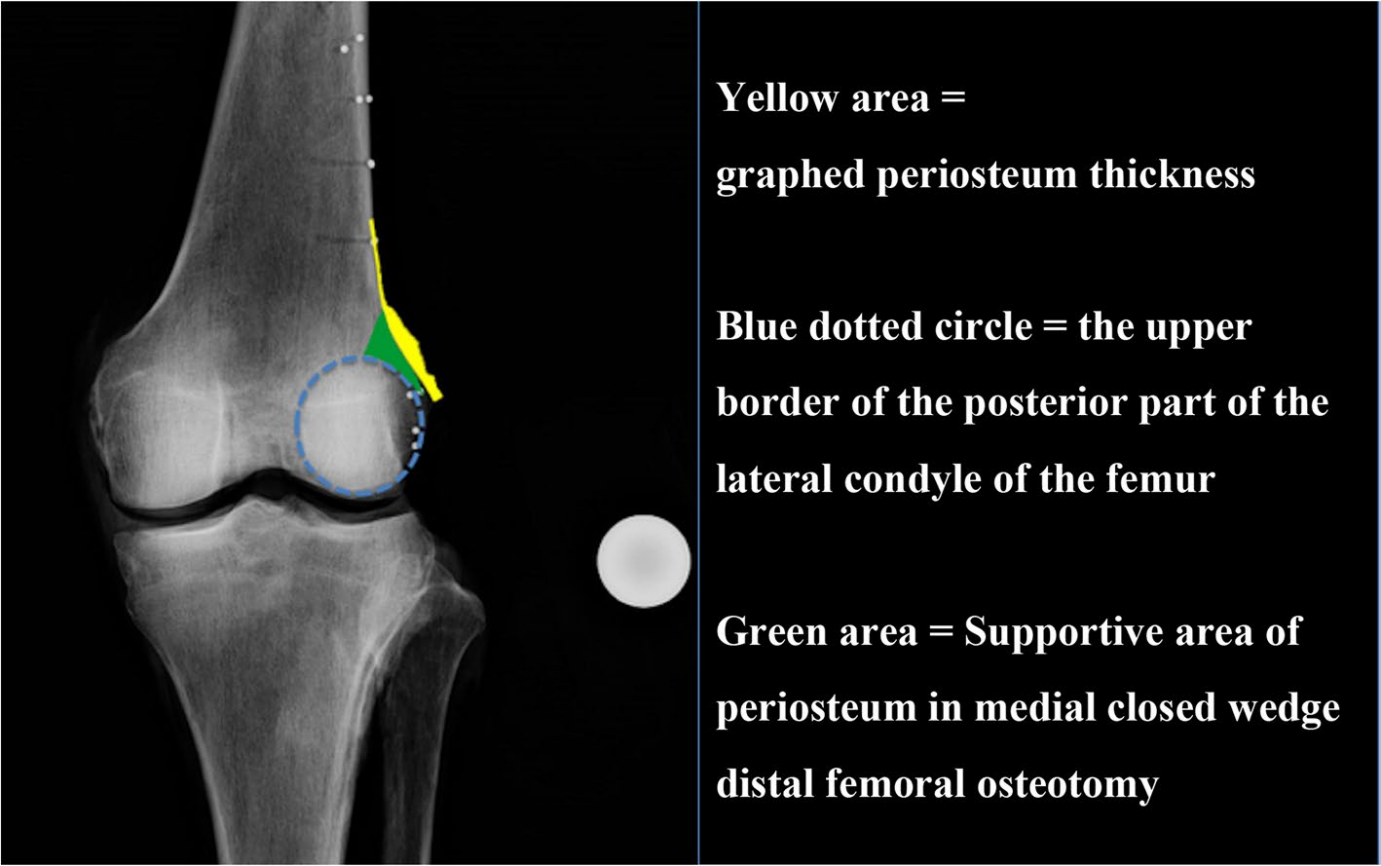
Supportive area of periosteum in medial closed wedge distal femoral osteotomy (MCWDFO). The periosteum might support the hinge of MCWDFO within the area surrounded by the bony landmark and the upper border of the posterior part of the lateral condyle of the femur

The most important finding of the present study is that the periosteal thickness changed at the region corresponding to the apex of the turning point of the femoral metaphysis in all cases. Therefore, the periosteum might support the hinge of MCWDFO within the area surrounded by the bony landmark and the upper border of the posterior part of the lateral condyle of the femur (Fig. 11).

Our pilot study showed the presence of the GLH on the posterolateral side of the distal femur. A previous cadaveric study of 20 human knees showed that the upper and lower margins of the femoral attachment of the GLH were 9.1 ± 0.9 mm above and 8.0 ± 1.4 mm below the upper border of the posterolateral region of the lateral femoral condyle [9]. Similarly, the present study revealed that the GLH was attached to the posterolateral side of the femur; however, the GLH was attached distally and posteriorly to the hinge and seemed to play a small role in stabilizing the hinge point. In contrast, another study revealed a different origin of the GLH [16], indicating the presence of morphological diversity and the need for further research.

Dissection of the lateral distal femur revealed the presence of the joint capsule and periosteum directly attached at the lateral side of the distal femur. A previous cadaveric study of 20 fresh-frozen human knees showed minimal soft tissue attachment around the hinge point of MCWDFO [12]. However, despite the presence of thick periosteum in the distal femur, the authors concluded that the soft tissue plays only a small supportive role in the stabilization of the osteotomy hinge, although no specific reason or details were given [12]. The radiological analysis revealed that the joint capsule was only partially attached to the hinge point of MCWDFO and was fragile enough to be easily detached during dissection. Furthermore, histological examination showed sparse fibrofatty tissue between the synovial surface of the joint cavity and the periosteum; thus, the joint capsule seemed to play a small role in stabilizing the hinge point. In contrast, thick periosteum was present around the metaphysis region, which may provide additional support to the osteotomy hinge.

The mean periosteal thicknesses of the metaphysis and diaphysis in the present study were similar to those in a cadaveric study of 18 femoral diaphyses, which revealed thicknesses of the cambium and fibrous layers of 23 ± 2.5 μm and 77 ± 8.8 μm, respectively [19]. No previous anatomical study has revealed the periosteal thickness of the distal femur, especially around the hinge point of MCWDFO. The present study is the first to compare the periosteal thickness between the metaphysis and diaphysis of the femur. Periosteal expansion occurs throughout life. The rate of expansion is fastest in puberty and becomes slower in adulthood [20, 21]. However, the decreased serum estradiol levels in postmenopausal women are correlated with changes in the periosteal diameter, and the rate of expansion accelerates again after menopause [22]. In the present study, the periosteal thickness in the region of the diaphysis was also significantly correlated with age; a similar tendency was observed in the region of the metaphysis, but there was no significant correlation. Sex-related differences could not be investigated because of the small sample size.

The periosteum also thickens in response to mechanical stress from components of the enthesis, such as ligaments, tendons, and muscles, and the resultant expansion of the periosteum increases the strength of bone and decreases the risk of fracture [23, 24]. In the present study, a strong correlation was observed between the periosteal thickness and the AP dimension of the femur, the total length of the femur, and height. Generally, increases in these measurements are directly proportional to the mechanical stress and load. However, it has also been reported that the periosteal thickness increases as a compensatory response to osteoporotic bone [24]. Thus, it should be emphasized that the thickness of the periosteum does not necessarily guarantee the mechanical stability of the structure, including the bone and further study is needed to understand the effect of periosteal thickness on mechanical strength.

The graphically visualized results of the periosteal thickness in the present study showed that the periosteal thickness of the distal lateral femur tended to rapidly decrease from the metaphyseal region toward the diaphyseal region. Furthermore, the overlayed images showed that the periosteal thickness changed at the part corresponding to the apex of the turning point of the femoral metaphysis in all cases. These alterations might be explained by the differences in bony geometry between the metaphysis and diaphysis and the difference in the soft tissue structure surrounding these bones. The bony landmark corresponding to the apex of the turning point of the femoral metaphysis is clinically important because it is easy to recognize and can be referred to intraoperatively during osteotomy as well as during preoperative planning and postoperative evaluation.

The present study has several limitations. First, the mean age of the cadavers was 78.8 ± 9.3 years (range, 61–91 years), which was relatively older than the typical patient undergoing MCWDFO. However, age seemed to have little influence on the anatomical location of the joint capsule attachment or the morphological features of the turning point of the periosteal thickness. Second, the cadavers had been fixed with formalin. The strength of the periosteum was not evaluated because of the formalin fixation of the cadavers. Nevertheless, this is the first report of quantitative measurements of the periosteal thickness around the hinge point of MCWDFO. Similarly, although the joint capsule was fragile enough to be easily detached during dissection, changes in mechanical features associated with tissue fixation were not considered. Third, the area of marked capsule attachment around the ideal hinge point of MCWDFO was 206.8 ± 31.7 mm^2^ (range, 164.3–278.1 mm^2^). Further investigations of the capsule attachments at other sites are needed to describe the clinical significance of this result. However, as already mentioned, the joint capsule seemed to play a small role in stabilizing the hinge point based on the anatomical and histological results. Fourth, a detailed anatomical evaluation of the femoral attachment of the GLH was not performed. The present study revealed that the GLH was attached distally and posteriorly to the hinge and seemed to play a small role in stabilizing the lateral hinge point. However, descriptions of the attachment site of the GLH differ among various reports. Therefore, when lateral hinge fracture occurs, the GLH may have a mechanical effect on the fracture site (such as flexion dislocation of the fractured hinge through ankle plantar dorsiflexion) depending on its posterior femoral attachment site. Fifth, we investigated the soft tissue only from the lateral epicondyle to the proximal direction; that on the distal side of the lateral epicondyle remains unclear. A previous cadaveric study of fresh-frozen human knees showed that the fibular collateral ligament had an average femoral attachment site slightly proximal (1.4 mm) and posterior (3.1 mm) to the lateral epicondyle. Moreover, the popliteus tendon attached anterior and inferior to the fibular collateral ligament, and the average distance between the femoral attachments of the popliteus tendon and fibular collateral ligament was 18.5 mm [16]. However, in the present study, histological examination revealed that only the capsule and periosteum were present around the hinge point of MCWDFO. Additionally, the risk of cutting into the joint cavity increases when the hinge is made on the more distal side of the lateral epicondyle, and no reports to date recommend such distal-level osteotomy as a safe zone. Lastly, this was a 2D study and did not include 3D structural imaging of the hinge point or the femoral bone morphology. Therefore, further study is needed based on these findings of this present study.

## Conclusions

In conclusion, the periosteum may support the hinge of MCWDFO within the area surrounded by the apex of the turning point of the femoral metaphysis and the upper border of the posterior part of the lateral femoral condyle.

## Data Availability

The datasets collected and/or analyzed during the current study are available from the corresponding author on reasonable request.

## Abbreviations

MCWDFO: Medial closed wedge distal femoral osteotomy
GLH: Gastrocnemius lateral head
ML: Mediolateral
AP: Anteroposterior
HE: Hematoxylin and Eosin staining
CB: Cortical bone
PO: Periosteum
FA: Fibrous adipose tissue
MT: Masson's Trichrome stain
fib: Fibrous layer of the periosteum
cam: Cambium layer of the periosteum
